# Design of Iron(II) Phthalocyanine‐Derived Oxygen Reduction Electrocatalysts for High‐Power‐Density Microbial Fuel Cells

**DOI:** 10.1002/cssc.201700851

**Published:** 2017-08-01

**Authors:** Carlo Santoro, Rohan Gokhale, Barbara Mecheri, Alessandra D'Epifanio, Silvia Licoccia, Alexey Serov, Kateryna Artyushkova, Plamen Atanassov

**Affiliations:** ^1^ Department of Chemical and Biological Engineering Center for Micro-Engineered Materials, CMEM University of New Mexico Advanced Materials Lab 1001 University Blvd. SE Suite 103, MSC 04 2790 Albuquerque NM 87131 USA; ^2^ Department of Chemical Science and Technologies University of Rome Tor Vergata Via della Ricerca Scientifica 00133 Rome Italy

**Keywords:** carbon, energy storage, iron, microbial fuel cells, oxygen reduction reaction

## Abstract

Iron(II) phthalocyanine (FePc) deposited onto two different carbonaceous supports was synthesized through an unconventional pyrolysis‐free method. The obtained materials were studied in the oxygen reduction reaction (ORR) in neutral media through incorporation in an air‐breathing cathode structure and tested in an operating microbial fuel cell (MFC) configuration. Rotating ring disk electrode (RRDE) analysis revealed high performances of the Fe‐based catalysts compared with that of activated carbon (AC). The FePc supported on Black‐Pearl carbon black [Fe‐BP(N)] exhibits the highest performance in terms of its more positive onset potential, positive shift of the half‐wave potential, and higher limiting current as well as the highest power density in the operating MFC of (243±7) μW cm^−2^, which was 33 % higher than that of FePc supported on nitrogen‐doped carbon nanotubes (Fe‐CNT(N); 182±5 μW cm^−2^). The power density generated by Fe‐BP(N) was 92 % higher than that of the MFC utilizing AC; therefore, the utilization of platinum group metal‐free catalysts can boost the performances of MFCs significantly.

## Introduction

Microbial fuel cells (MFCs) are very attractive bioelectrochemical systems capable of degrading and removing organic pollutants and generating electricity.^1^, ^2^, ^3^ To be competitive with existing wastewater‐treatment approaches, the pollutant‐removal efficiency has to be increased considerably; therefore, the kinetics of water purification should be accelerated. In general, the power/current produced in MFCs is quite low, the electrochemical processes require substantial optimization, and, in parallel, the losses associated with these processes have to be reduced.^4^, ^5^ To date, several successful prototypes and scaled‐up systems have been presented to demonstrate the potential of MFC technology for both wastewater treatment and clean‐energy generation.^1^, ^2^, ^6^, ^7^

It also should be mentioned that major problems associated with the performances of MFC systems originate from the poor kinetics for the oxygen reduction reaction (ORR) and the high overpotentials of the cathode operating in neutral media.^4^, ^8^, ^9^ Oxygen is primarily used as an electron acceptor in the majority of fuel cells as it is naturally available in the atmosphere (and, consequently, has a low cost) and has a high redox potential. An additional performance drop of MFCs is related to the fact that oxygen electroreduction requires H^+^ or OH^−^ ions as reagents (depending on the mechanism of the ORR), and their concentration is lowest at neutral pH (≈10^−7^ m). Therefore, the ORR is severely hampered, which negatively affects the overall MFC performance. The general practice to reduce the overpotentials and accelerate the kinetics is utilization of electrocatalysts on the cathode.^10^, ^11^, ^12^, ^13^, ^14^

A literature review indicates three main types of catalysts that can be integrated into the cathodic structures of MFCs: (i) platinum‐group metals (PGMs),^10^, ^11^, ^12^ (ii) carbonaceous metal‐free catalysts,^10^, ^11^, ^12^ and (iii) PGM‐free materials.^10^, ^11^, ^12^ The first type of catalyst utilizes platinum/PGM nanoparticles dispersed on carbonaceous supports and can be used conventionally as anode and cathode catalysts in hydrogen–air or direct‐alcohol fuel cells.^15^, ^16^, ^17^, ^18^, ^19^ Several issues are related to the employment of Pt as a cathode catalyst in MFCs. First, Pt is a rare and very expensive metal, and large‐scale deployment for practical applications seems unviable and cost‐prohibitive owing to the low power produced by the MFCs.^20^ Second, MFCs work in harsh and polluted environments in which platinum will interact with strongly adsorbed charged or neutral species, which will lead to a decrease in ORR catalytic activity.^21^, ^22^, ^23^, ^24^ For example, Cl^−^ and S^2−^ ions are such species, and small concentrations of them can poison the platinum surface and decrease its ORR activity.^21^, ^22^, ^23^, ^24^ Despite the fact that Pt is the most common catalyst for MFCs, it has unavoidable issues with durability, and alternative electrocatalysts should be deployed.^10^, ^25^

Carbonaceous materials can be considered as a suitable replacement for Pt catalysts^10^, ^11^, ^12^ because they possess unique characteristics such as their high mechanical strengths, resistance to corrosion, high conductivities, and high surface areas together with moderately good ORR activity in neutral media.^10^, ^11^, ^12^ Their low cost and broad commercial availability are also important factors that make them appropriate for large‐scale applications. In 2009, it was shown that activated carbon integrated into an MFC cathode can achieve a substantially high electrochemical performance.^26^ Since this pioneering study, activated carbon has been used frequently as a cathode catalyst in different types of MFCs.^27^, ^28^, ^29^, ^30^, ^31^ It should be noted that other carbonaceous materials such as carbon nanotubes,^32^ activated carbon fibers,^33^ modified carbon black,^34^ and graphene^35^, ^36^, ^37^, ^38^ were also studied as cathode catalysts in MFCs. On the other hand, systematic research into carbonderived cathodes revealed intrinsic low ORR activities, high overpotentials, and low power generation.

Recently, PGM‐free materials have been studied comprehensively as electrocatalysts for oxygen reduction in different types of fuel cells. Among these PGM‐free catalysts, M−N−C (M=transition metal) materials are most active in oxygen electroreduction in acidic and alkaline media. For M−N−C catalysts, there are several synthesis methods, which can be divided into two categories. The first and most common method is based on the high‐temperature treatment (pyrolysis) of a metal salt and organic precursors rich in nitrogen and carbon (N−C precursors).^39^ For the last ten years, earth‐abundant transition metals such as Co, Mn, Ni, and Fe were used for the preparation of M−N−C‐type catalysts.^40^, ^41^ Catalysts containing the same organic precursors and different metals have been fabricated and tested using rotating ring disk electrodes (RRDEs) in neutral media. Several research groups found that Fe‐based catalysts outperformed Co‐, Mn‐, and Ni‐ based catalysts and also showed high durability over 10 000 cycles.^42^ The majority of the studied M−N−C electrocatalysts can reduce oxygen through a 2×2e^−^ transfer mechanism.^42^ The catalysts were incorporated into air‐breathing cathodes and tested in MFCs, and Fe−N−C catalysts were superior to those containing other metals.^43^, ^44^, ^45^ Multiple Fe−N−C catalysts were synthesized from different N−C precursors; their ORR performances were correlated with the density of the different active sites formed on the surface of the carbon matrix.^46^ In general, the performances increased linearly with the metal‐coordinated, pyridinic, and pyrrolic nitrogen content.^46^ The reverse relationship was identified between performance and graphitic nitrogen atoms.^46^ Several examples of M−N−C catalysts with M as Co,^47^, ^48^, ^49^ Ni,^50^, ^51^ Mn,^52^, ^53^, ^54^ Fe,^22^, ^23^, ^43^, ^44^, ^45^, ^46^, ^55^, ^56^, ^57^ and other metals^58^, ^59^ were reported.

The second method for the preparation of PGM‐free catalysts is based on the modification of carbonaceous supports such as carbon black (CB), carbon nanotubes (CNTs), or graphene with metal complexes such as Fe or Co porphyrins and phthalocyanines with preformed Fe−N_*x*_ centers.^39^, ^44^, ^52^, ^57^, ^60^, ^61^, ^62^, ^63^ This method of ORR catalyst design has been discussed in several articles^39^,^44^; the metal centers bound to the nitrogen atoms in the complex act as the active sites, whereas the conductive carbonaceous support improves the electron transfer.^39^,^44^ These materials are very active towards the ORR but do not show high stability under different pH conditions. To increase their stabilities, these hybrids can be pyrolyzed, and this approach is close to the first method described above.

In this study, Fe‐based catalysts were fabricated from commercially available iron(II) phthalocyanine, which was tethered to the surfaces of two different carbonaceous supports, that is, (i) nitrogen‐doped multi‐walled carbon nanotubes and (ii) nitrogen‐doped carbon black (Black Pearls) to produce Fe‐CNT(N) and Fe‐BP(N), respectively. The surface chemistry and morphology of the obtained materials was studied comprehensively. The electrocatalytic activities of these materials in the ORR were studied by employing the RRDE method. Finally, the catalysts were incorporated into air‐breathing cathodes and tested in operating MFCs. The performances of the new catalysts were compared to that of activated carbon (AC) as a carbonaceous benchmark.

## Results and Discussion

### Surface morphologies of the catalysts

**Figure 1:**
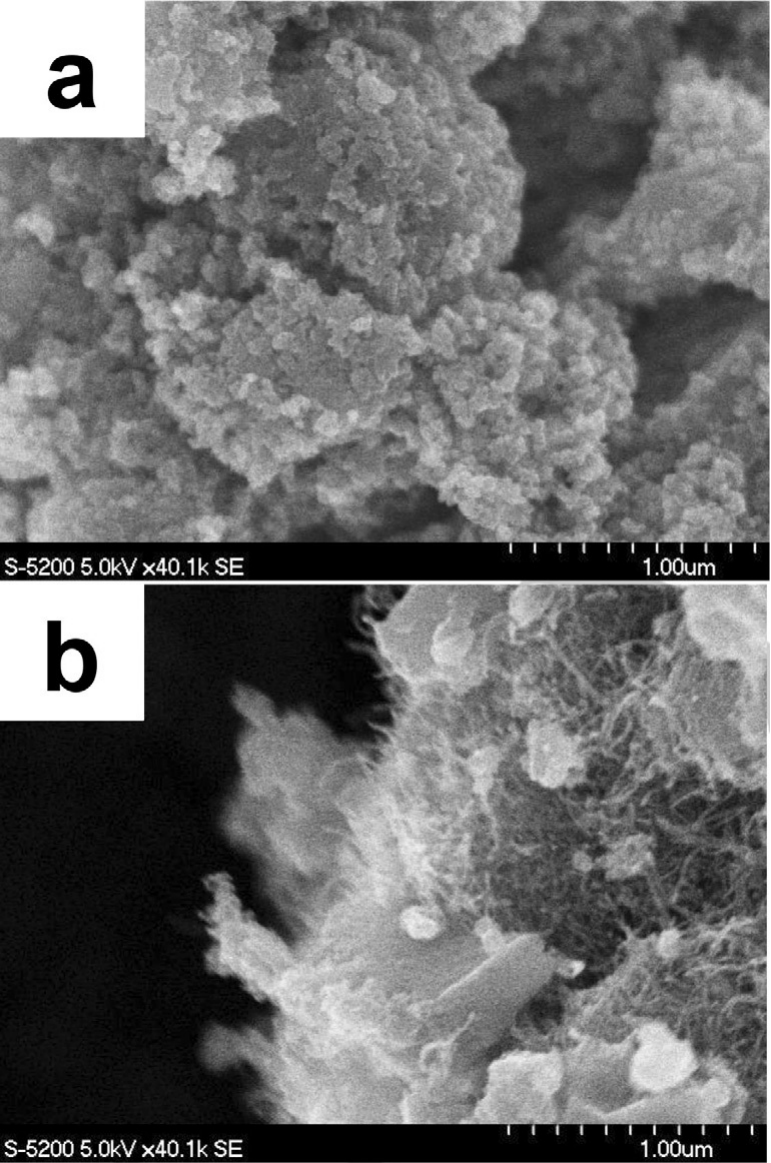
SEM images of a) Fe‐BP(N) and b) Fe‐CNT(N).

**Figure 2:**
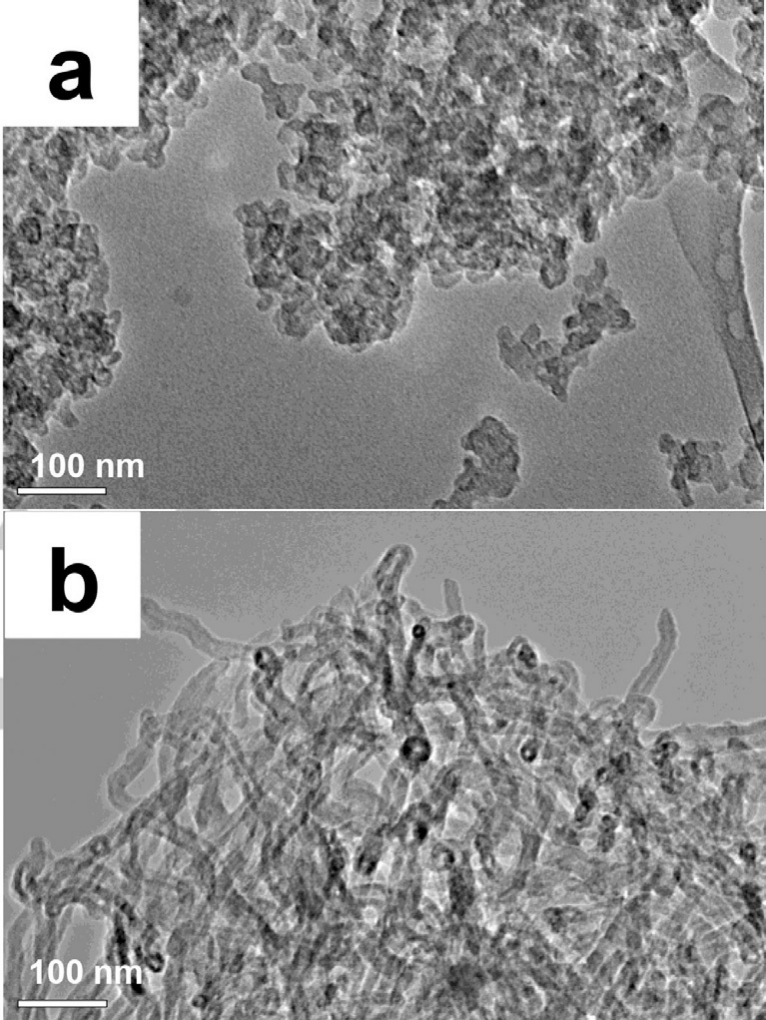
TEM images of a) Fe‐BP(N) and b) Fe‐CNT(N).

The morphological features of the two catalysts used in this work were studied by electron microscopy (Figures 1 and 2). The SEM image (Figure 1 a) of the Fe‐BP(N) catalyst shows the presence of micrometer‐sized agglomerated Black‐Pearl particles (carbon spheres). The higher‐magnification TEM image (Figure 2 a) of the Fe‐BP(N) catalyst demonstrates the agglomeration of the carbon spheres (with a primary carbon particle size of 10–50 nm).

The multi‐walled carbon nanotube‐based catalyst [Fe‐CNT(N)] exhibits a clearly different morphology compared with that of the Fe‐BP(N). The SEM image of the Fe‐CNT(N) catalyst (Figure 1 b) reveals the presence of large individual, micrometer‐sized crystallites. However, resolution imaging further reveals the presence of a large number of carbon nanotubes in the nanostructure of the crystallites. The outer coating of this structure appears to be graphitic in nature. Thus, the entire morphology of the material appears to be an outer graphitic carbon coating supported on a carbon nanotube assembly. The TEM images (Figure 2 b) of the Fe‐CNT(N) catalyst further confirm the presence of the carbon nanotube assembly in the structure.

### Surface chemistry of the catalysts

The surface chemistry of the two catalysts was investigated through X‐ray photoelectron spectroscopy (XPS). The elemental compositions and chemical speciations are shown in Table 1. The catalysts mainly consist of carbon, the concentration of which varies from 79.1 % to 81.9 %. The oxygen content was noticeably high, with total percentage varying from 9.3 % to 11 %. The nitrogen content varies from 5.8 % for Fe‐BP(N) to 10.0 % for Fe‐CNT(N).

**Table 1:** Elemental compositions and chemical speciations of the two catalysts of interest.

Sample	Peak	C species^[a]^ [%]			Peak	Peak	N species [%]				Peak	Fe species^[b]^ [%]	
	C 1s [%]	C_graphitic_	C−C, C*	C_*x*_O_*y*_	O 1s [%]	N 1s [%]	N_pyr._	N−Fe	N−H	N ^gr.^	Fe 2p [%]	Fe−N	Fe_*x*_O_*y*_
Fe‐BP(N)	81.9	8.1	46.7	44.6	11	5.8	62.2	26.7	10.7	0.4	1.3	8.2	91.8
Fe‐CNT(N)	79.1	9.8	24.8	61.9	9.3	10.0	47.4	28.3	18.0	6.2	1.5	6.8	93.2

Nitrogen has been identified to have a positive effect on the ORR owing to its ability to improve the electronic properties.^64^, ^65^, ^66^ However, excess nitrogen concentrations may be harmful to the overall performance, as it decreases the conductivity of the material. Very high Fe contents between 1.3 % and 1.5 % were also identified.

**Figure 3:**
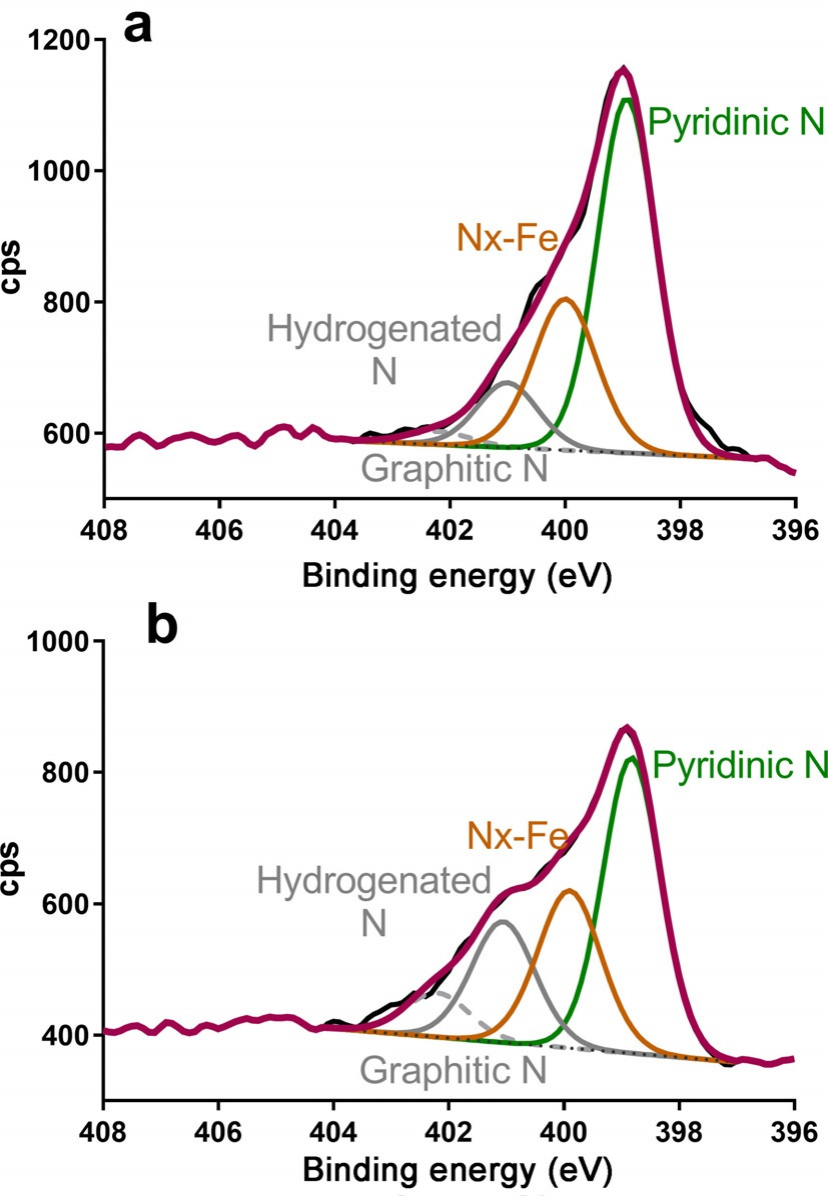
High‐resolution N 1s spectra of a) Fe‐BP(N) and b) Fe‐CNT(N).

In a previous study, pyridinic nitrogen and transition‐metal‐coordinated nitrogen atoms showed a positive relationship with performance.^42^ The high‐resolution N 1s spectra for the two catalysts are shown in Figure 3 and were fitted with four peaks, namely, pyridinic nitrogen atoms (N_pyr._) at a binding energy of 398.5 eV, Fe‐coordinated nitrogen atoms (N−Fe) at 399.5 eV, hydrogenated nitrogen atoms (N−H, pyrrolic nitrogen and hydrogenated pyridine) at 401 eV, and graphitic nitrogen atoms (N_gr._) at 402.3 eV. For the two materials tested, the relative percentage of pyridinic nitrogen atoms was the highest of all types of nitrogen atoms and varied from 47.4 % to 62.2 %, and the Fe‐BP(N) sample contained the largest amount. Importantly, pyridinic nitrogen atoms represent an edge defect within the carbon matrix, and edge sites are more favorable for oxygen reduction. The Fe‐CNT(N) catalyst had the larger relative percentages of hydrogenated nitrogen and Fe‐coordinated N atoms of 18.0 % and 28.3 %, respectively. The peak at 399.5 eV is assigned to Fe‐coordinated nitrogen atoms and may also have a contribution from amines. The analysis of the high‐resolution Fe 2p spectra reveals that the well‐resolved peak for the Fe−N active centers is larger for the Fe‐BP(N) sample. The Fe‐CNT(N) samples has a substantially larger amount of hydrogenated nitrogen atoms. The negative effect of hydrogenated nitrogen atoms on the ORR due to its significant contribution to the reduction of oxygen to hydrogen peroxide was shown previously.^67^ The Fe‐BP(N) catalyst also has the smallest amount of graphitic carbon atoms with the majority being amorphous with surface oxides, which are an important marker for a large number of defect sites within the carbon network, and this is related to a higher density of active ORR sites.^67^ The combination of higher amounts of nitrogen edge defects, higher amount of iron‐coordinated nitrogen atoms, and higher amount of aliphatic carbon atoms and surface oxides should make the Fe‐BP(N) sample more effective in the ORR.

### Electrocatalytic activities of the catalysts in neutral media

RRDE measurements were performed using the three catalysts in an O_2_‐saturated electrolyte to evaluate the oxygen reduction activity as well as the total number of electrons transferred in the electrocatalytic process [Eq. (1)] and the hydrogen peroxide generated [Eq. (2)]. Two different catalyst loadings (0.1 and 0.6 mg cm^−2^) were used for the experiments. It was shown previously that an increase in loading could hinder the catalyst kinetics.^38^, ^68^, ^69^

**Figure 4:**
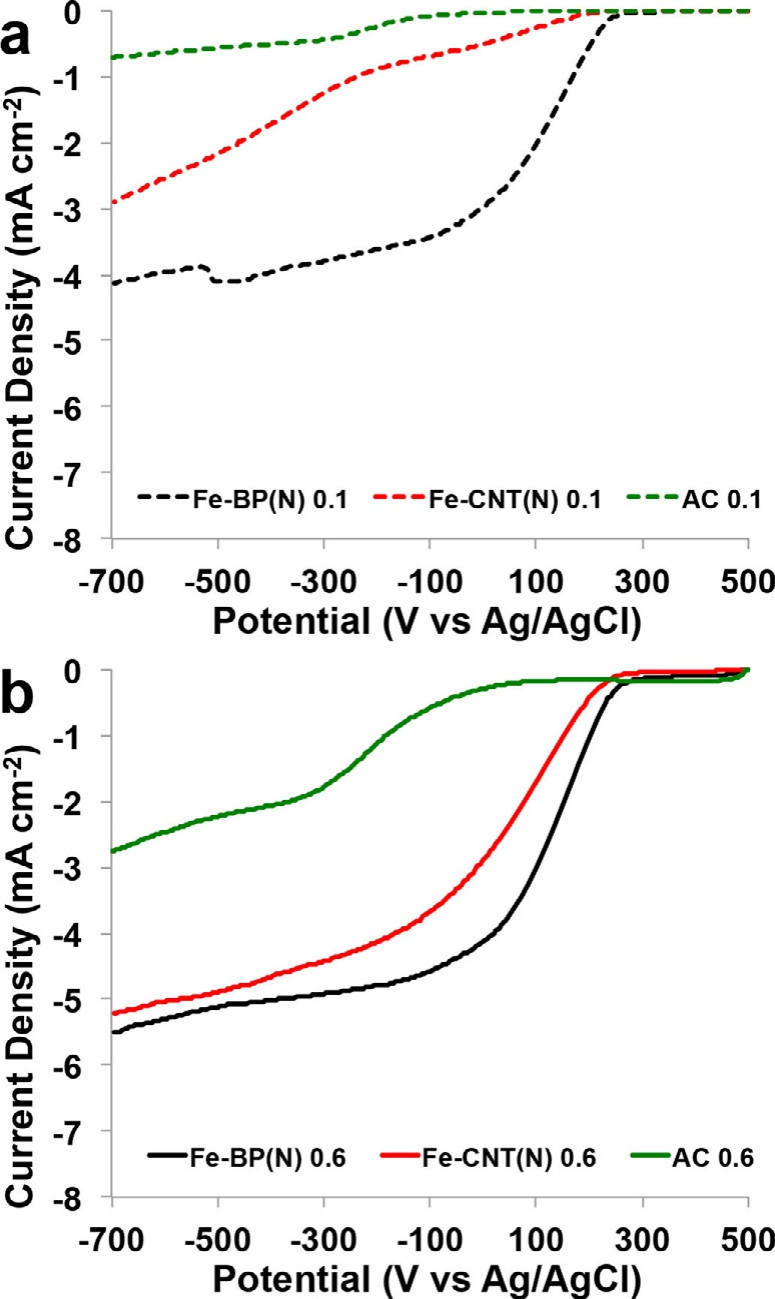
RRDE data for catalysts with different loadings of a) 0.1 and b) 0.6 mg cm^−2^.

**Figure 5:**
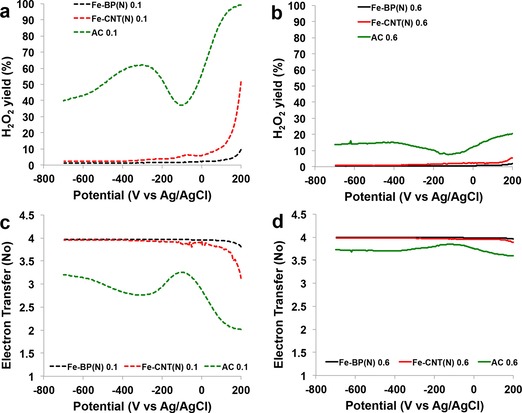
H_2_O_2_ yields at loadings of a) 0.1 and b) 0.6 mg cm^−2^; electron transfer numbers at loadings of c) 0.1 and d) 0.6 mg cm^−2^

A thick catalytic layer of porous carbonaceous material can trap the intermediate peroxide inside the pores, where the H_2_O_2_ is consumed (either by chemical decomposition or by electrochemical reduction to water) without being detected on the ring.^38^, ^68^, ^69^ As the material loading on the disk increased, the peroxide detected decreased significantly.^38^, ^68^, ^69^ Therefore, two extreme loadings, one low (0.1 mg cm^−2^) and one high (0.6 mg cm^−2^), were selected to obtain reasonable data for the ORR with these catalysts. The Fe‐BP(N) catalyst exhibits a better performance than Fe‐CNT(N) as it has a higher onset potential, more positive half‐wave potential, and higher limiting current (Figure 4 a and b). Both of the Fe‐based catalysts exhibited a performance superior to that of AC. A similar trend was observed for both catalyst loadings. The peroxide percentage generated in the ORR with the three catalysts is consistent with the findings of the disk‐current measurements (Figure 5 a and b). The Fe‐BP(N) catalyst produced the least peroxide (≈1 %, Figure 5 a), and the Fe‐CNT(N) catalyst produced slightly more peroxide (≈1–2 %, Figure 5 a and b). Interestingly, the overall peroxide production is quite low for the FePc‐derived catalysts. The AC produced a much higher peroxide yield, in agreement with previously reported data (Figure 5 a and b). It was proven in this study that an increased loading leads to a significant decrease in detected peroxide (Figure 5 a and b). The Fe‐based catalysts exhibit a four‐electron reduction process independent of the loading (Figure 5 c and d). On the contrary, AC followed a two‐electron‐transfer mechanism at a low loading of 0.1 mg cm^−2^ (Figure 5 c). The thick layer at 0.6 mg cm^−2^ loading masked the AC behavior, and the electron transfer could be considered to be closer to a four‐electron process. It is important to mention here that the FePc catalysts greatly outperform commercial activated carbon in terms of ORR activity and, therefore, are suitable substitutes for AC for the ORR in neutral media.

### Power generation of operating MFCs

**Figure 6:**
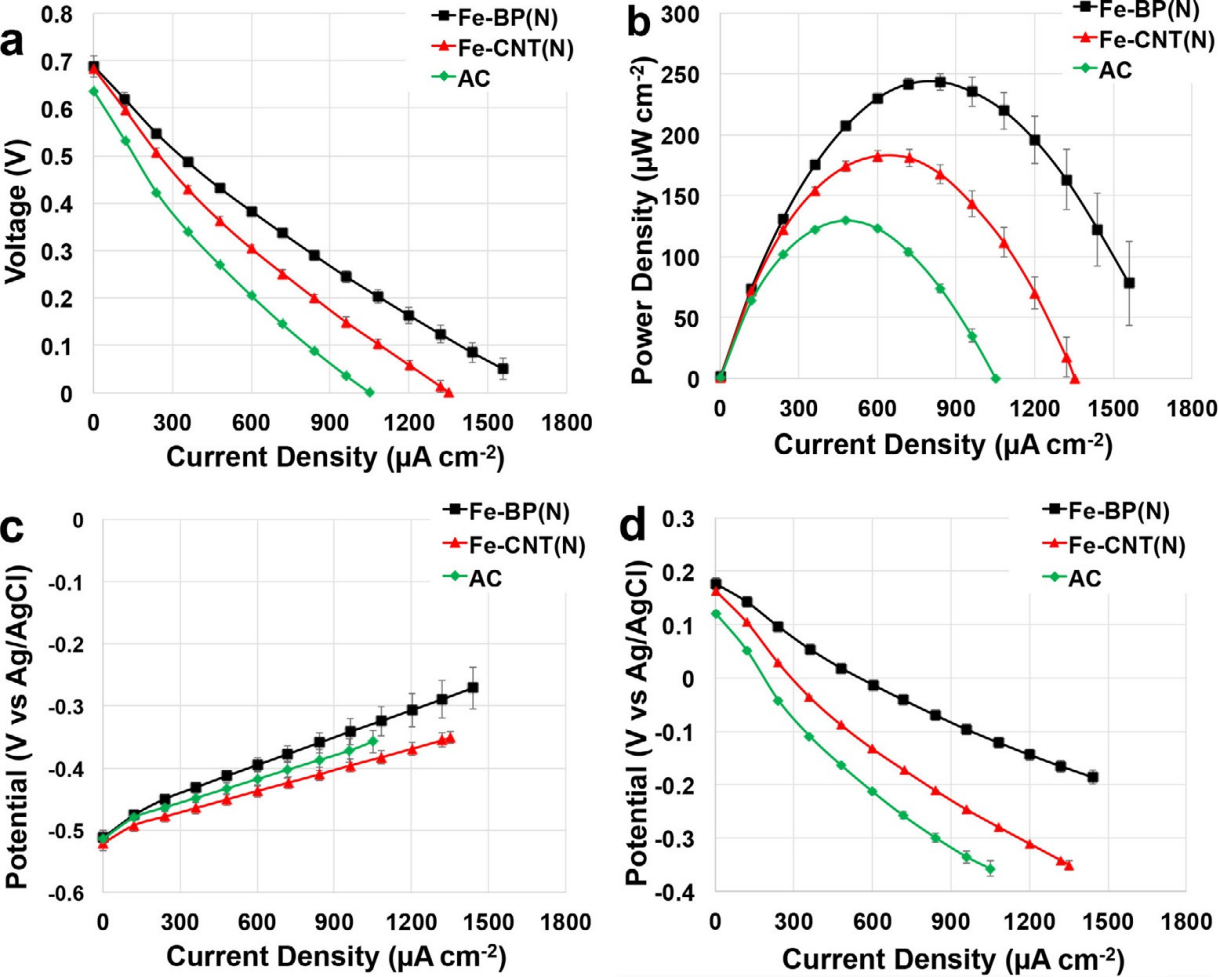
a) Overall polarization curves, b) power curves, c) anode polarizations, and d) cathode polarizations for Fe‐BP(N), Fe‐CNT(N), and AC.

Polarization curves were recorded for the different Fe‐based catalysts incorporated into an air‐breathing cathode composed of a carbonaceous matrix made of AC, CB, and polytetrafluoroethylene (PTFE) as the binder (Figure 6 a). The open‐circuit voltages (OCVs) measured at the beginning of the polarization curves were different for the materials investigated. The AC had the lowest OCV of (635±2) mV. The Fe‐BP(N) and Fe‐CNT(N) had higher OCV values of (688±12) and (684±8) mV, respectively (Figure 6 a). In the polarization curves, three distinctive trends can be noticed: AC has the poorest performance, Fe‐CNT(N) performs better than AC but worse than Fe‐BP(N), and Fe‐BP(N) has the best performance of the catalysts investigated (Figure 6 a). The highest power density measured in this investigation was that produced by Fe‐BP(N) of (243±7) μW cm^−2^ (Figure 6 b). Fe‐CNT(N) produced a lower power density of (182±5) μW cm^−2^ (Figure 6 b). The AC sample had the lowest power density of (127±1) μW cm^−2^ (Figure 6 b). The Fe‐BP(N) performed 33 % better than Fe‐CNT(N) and 92 % better than AC. The separate anode (Figure 6 c) and cathode (Figure 6 d) profiles show similar anodic performances, which underlines that the difference in the overall polarization curve was caused substantially by the cathode behavior.

### Outlook and comparison with existing literature

In MFC systems, the ORR is often identified as the most problematic aspect; and therefore solutions have to be investigated and considered.^4^, ^8^, ^9^ PGM‐free catalysts seem to be interesting and appropriate for further investigations. Once again, in this study, the utilization of PGM‐free catalysts resulted in a substantial increase in the power produced compared with that for AC. Among the earth‐abundant metals, Fe was selected for the catalyst because it was previously identified to be more active than other earth‐abundant metals such as Mn, Cu, Co, and Ni.^10^, ^11^, ^12^, ^13^ This work confirms that PGM‐free catalysts based on iron can boost the performance considerably. The power density was doubled for Fe‐BP(N) compared with that of bare AC [(243±7) and (127±1) μW cm^−2^, respectively]. The results were consistent for the data obtained during the RRDE tests and the data acquired during the MFCs tests. Therefore, it is demonstrated once more that RRDE data can be used to predict the performance of a catalyst incorporated into an air‐breathing cathode, in agreement with previously reported data.^46^

Different PGM‐free catalysts incorporated into air‐breathing cathodes were reported previously.^55^, ^70^, ^71^, ^72^, ^73^ Yang et al. integrated Co/N−C nanoparticles in an air‐breathing cathode and obtained a maximum power density of 251 μW cm^−2^.^70^ The same research group also utilized a NiCo_2_O_4_‐modified activated‐carbon cathode and obtained a lower maximum power density of 173 μW cm^−2^.^71^ Fu et al.,^72^ Pan et al.,^73^ and Yang and Logan^55^ decorated activated‐carbon‐based cathodes with Fe‐based catalysts and obtained maximum power densities of 143, 244, and 260 μW cm^−2^, respectively, with an electrolyte containing 50 mm phosphate buffer, acetate as bacterial food, and MFCs operating at 30 °C.

Compared to previously reported studies, the main feature of this work is the utilization of Fe‐based catalysts that are fabricated through the attachment of commercially available iron(II) phthalocyanine onto high‐surface‐area carbon–nitrogen‐doped supports instead of a high‐temperature pyrolysis approach. The two carbonaceous supports selected were nitrogen‐doped carbon nanotubes [CNT(N)] and nitrogen‐doped Black Pearls [BP(N)]. The positive effect of nitrogen on the ORR performances was elucidated previously.^22^, ^23^, ^46^ BP(N) was the support with the highest BET surface area among the materials used in this study; the previously quantified BET surface area of 1317 m^2^ g^−1^ is almost four times higher than that for CNT(N) of 359 m^2^ g^−1^.^57^ The BP(N) also showed the best performances in the RRDE and MFC tests [(243±7) μW cm^−2^].

To be suitable for large‐scale applications, Fe‐based catalysts must also be cheap and durable. Many parameters will affect the final price of PGM‐free electrocatalysts, and economical price analysis is generally quite complicated. In a previous study, the cost of the catalyst produced through the sacrificial‐support method and fabricated through a pyrolysis technique was quantified as approximately 3.5 US$ g^−1^.^25^ The estimation only considered the materials utilized; unfortunately, the gas utilized during pyrolysis was not included and, most importantly, the cost of the electricity utilized during the heat treatment was not considered. High‐temperature processes are very energy consuming as electricity is used to keep the temperature within the furnace constant during the pyrolysis process. The costs of the catalysts on the basis of the lab‐scale procedure utilized were estimated to be 8 and 5 US$ g^−1^ for Fe‐CNT(N) and Fe‐BP(N), respectively. This estimation seems to be more realistic, as the procedure presented in this manuscript does not have high‐temperature processes, and this leads to an overall reduction of the preparation costs that should be taken into serious consideration. Moreover, as the heat treatment is avoided, the fabrication of the catalysts is simpler and more affordable.

The maximum power density obtained in this work was (243±7) μW cm^−2^ for the BP(N) support. A direct comparison with previously reported materials is difficult owing to the different operating conditions utilized. The performance is effected dramatically by the MFC design,^1^ operating temperature,^74^ altitude above sea level, electrolyte utilized (different solution conductivity),^43^ bacteria utilized (e.g., single or mixed culture),^1^ organic compounds utilized (e.g., lactate, acetate, fumarate, etc.),^3^, ^75^ organics concentration,^74^ and the presence or absence of membranes.^66^ Several other studies demonstrated the utilization of Fe‐based catalysts with excellent performances and superiority over platinum‐based cathodes^22^, ^23^ or AC‐based cathodes.^43^

The power densities usually vary between approximately 100 and 600 μW cm^−2^.^76^, ^77^ The highest values were obtained with an electrolyte with a high solution conductivity and large anodes.^43^, ^55^, ^78^, ^79^ Even higher power densities of up to 600 μW cm^−2^ were reported for Pt‐based cathodes but for very short times.^80^

A direct comparison can instead be made with previously presented work in which an identical (i) MFC configuration (single‐chamber MFC), (ii) operating temperature [(22±2) °C], (iii) altitude above sea level [experiments conducted in Albuquerque, New Mexico, at 1500 m above mean sea level (AMSL)], (iv) electrolyte (50 % activated sludge, 50 % 0.1 m K‐PB (potassium phosphate buffer), and 0.1 m KCl (potassium chloride)), (v) mixed‐culture bacteria, (vi) organic compound (sodium acetate), and (vii) membrane‐less configuration were utilized.^22^, ^23^, ^38^, ^43^, ^46^ The obtained maximum power density achieved [(243±7) μW cm^−2^] is one of the highest power densities recorded under the same operating conditions and is second only to that of (251±2) μW cm^−2^ for Fe‐AAPyr (AAPyr=aminoantipyrine).^43^ The difference was just 3 % and, therefore, the results can be considered to be comparable. This result is interesting because it indicates that Fe‐BP(N) has a high activity towards the ORR that is comparable with those of catalysts fabricated through high‐temperature pyrolysis. Further investigations should focus on the stabilities and durabilities of these catalysts under long‐term operation. This will certainly be an aspect that will be considered in future studies.

## Conclusions

New Fe−N−C catalysts were obtained through the deposition of iron(II) phthalocyanine on two different high‐surface‐area carbonaceous materials. These catalysts were fabricated without the utilization of high‐temperature pyrolysis methods and deposited on (i) carbon black (Black Pearls) doped with nitrogen [Fe‐BP(N)] and (ii) multi‐walled carbon nanotubes doped with nitrogen [Fe‐CNT(N)]. The catalyst kinetics was studied using a rotating ring disk electrode (RRDE), and the results showed the superiority of Fe‐BP(N) in terms of its onset potential, half‐wave potential, and limiting current. Fe‐CNT(N) showed much higher performances compared with that of activated carbon (AC), which was used as a control. The catalysts were then integrated into air‐breathing cathodes and tested in microbial fuel cells. Fe‐BP(N) had the highest power density output of (243±7) μW cm^−2^, which was over 90 % higher than that of AC [(127±1) μW cm^−2^].

## Experimental Section

### Preparation of N‐doped carbonaceous supports

Multi‐walled carbon nanotubes (CNTs) were purchased from Sigma–Aldrich, and Black Pearls 2000 (BP) were purchased from Cabot Corporation. The CNTs and BP were modified by a two‐step treatment with nitric acid and ammonia gas. In the first treatment, the materials were heated in concentrated HNO_3_ (65 wt %) under reflux at 90 °C for 16 h. Then, the materials were collected by filtration and washed with distilled water until neutral pH was obtained. The materials were then dried in an oven at 70 °C overnight and ground with an agate mortar and pestle. In the second treatment, a flow of anhydrous ammonia was fed into in a tubular oven at *T*=400 °C (heating rate 5 °C min^−1^) for 4 h. The obtained products were labeled as CNT(N) and BP(N).

### Deposition of the Fe catalyst on the carbonaceous supports

Iron(II) phthalocyanine (FePc, Aldrich; 0.5 g) was dispersed in methanol (30 mL), and CNT(N) or BP(N) (0.5 g) was added. The mixture was stirred for 30 min in a water bath at 70 °C to evaporate the methanol, and the resulting powder was dried completely in a vacuum oven at 70 °C for 3 h to obtain samples labeled as Fe‐CNT(N) and Fe‐BP(N).

### Catalysts surface chemistry and morphology

The surface chemistry of the catalyst was identified through high‐resolution XPS with a Kratos Axis Ultra DLD spectrometer. Three separate areas of the same sample were analyzed, and the average values are presented. The average values have an error of less than 0.1 %. The high‐resolution O 1s, N 1s, C 1s, and Fe 2p spectra were obtained without the need for charge neutralization with a 225 W AlK_α_ monochromatic X‐ray source. The acquired spectra were then processed with the CASAxps software.

The surface morphologies of the catalysts were investigated through SEM and TEM. The SEM images were recorded using a Hitachi S‐800 instrument at different magnifications. The TEM imaging was performed with a JEOL 2010 instrument with samples on a copper grid. For simplicity, an image for each sample at one magnification was shown as representative of the sample.

### Cathode preparation

An air‐breathing cathode configuration was adopted in this study. A mixture of activated AC, CB, and PTFE was blended in a grinder in a weight ratio of 70:20:10 %, respectively. The mixture was placed in a die pellet and pressed over a stainless‐steel mesh (McMaster, USA), which was used as the current collector. The loading of the mixture was 40 mg cm^−2^. A pure AC cathode was fabricated by the above method and used as a control. The cathodes containing Fe‐BP(N) and Fe‐CNT(N) were prepared by the same method with a catalyst loading of 2 mg cm^−2^.

### RRDE analysis

The RRDE technique was used to study the catalyst kinetics of Fe‐BP(N), Fe‐CNT(N), and AC. The catalyst (5 mg) was mixed with 0.5 wt % Nafion solution (FuelCellStore, USA; 150 μL) and deionized water/isopropyl alcohol (DI/IPA) in a 1:1 ratio (850 μL). The obtained suspension was sonicated at least three times to obtain a uniform dispersion. The obtained ink was drop cast on the disk of a glassy carbon working electrode. Two loadings (0.1 and 0.6 mg cm^−2^) were used for each catalyst. The tests were performed in a solution containing 0.1 m potassium phosphate and 0.1 m KCl. The solution simulates an electrolyte with circumneutral pH value. Before the experiments, the electrolyte was saturated with oxygen for at least 30 min. Linear sweep voltammetry was then run from +0.5 to −0.7 V (vs. Ag/AgCl) at a scan rate of 5 mV s^−1^. The RRDE setup guarantees the possibility of measuring the current density produced by the disk (*j*_D_) but also the current density of the ring (*j*_R_) to quantify the intermediate (H_2_O_2_) produced during the ORR. From *j*_D_ and *j*_R_, the number of electrons transferred (*n*) during the ORR can be calculated with Equation (1):

(1) $$n=\left|\frac{4\times j_{D}}{j_{D}-j_{R}}\right|$$

Therefore, the percentage of H_2_O_2_ produced during the ORR can be calculated using Equation (2):

(2) $$H_{2}O_{2}\left[\%\right]=\frac{4-n}{2}\times 100$$

### MFC construction and operation

After the catalyst had been incorporated into the air‐breathing cathode, the cathode was screwed on a lateral hole of a modified Pyrex bottle with a volume of 125 mL.^22^, ^23^ The cathode part containing the AC, CB, and PTFE pellet faced the liquid, and the current collector faced the air side. The geometric area of the cathode was 2.85 cm^2^. The chamber was filled with a solution containing 50 vol % 0.1 m K‐PB and 50 vol % activated sludge from the Albuquerque Southeast Water Reclamation Facility located in Albuquerque, New Mexico, USA. Precolonized and well‐working anodes were moved from existing and running MFCs into MFCs with new and fresh cathodes. The anodes consisted of two carbon brushes with titanium cores (Millirose, USA) and a diameter and height of 3 cm each. The anode area was decided to be much higher than the cathode area as the latter was the subject of the study. The MFCs were left at the OCV for at least 3 h until the output stabilized. Polarization curves were then recorded using two potentiostats (Biologic‐USA, USA). The first potentiostat was connected in a two‐electrode mode with the anode as the working electrode and the cathode as the counter electrode short‐circuited with the reference channel. The second potentiostat was set up just to read the potentials of the anode and cathode versus the reference electrode (Ag/AgCl 3 m KCl). The polarization curves gave voltage– current curves as output. The power was calculated as the product of voltage and current. The current and power densities were shown as a function of the geometric area of the cathode, which was 2.85 cm^2^.

## Conflict of interest

*The authors declare no conflict of interest*.
